# Dysregulated peripheral proteome reveals NASH-specific signatures identifying patient subgroups with distinct liver biology

**DOI:** 10.3389/fimmu.2023.1186097

**Published:** 2023-06-05

**Authors:** Natalie Stiglund, Hannes Hagström, Per Stål, Martin Cornillet, Niklas K. Björkström

**Affiliations:** ^1^ Center for Infectious Medicine, Department of Medicine Huddinge, Karolinska Institutet, Karolinska University Hospital, Stockholm, Sweden; ^2^ Department of Upper GI, Karolinska University Hospital, Stockholm, Sweden; ^3^ Department of Medicine Huddinge, Karolinska Institutet, Stockholm, Sweden

**Keywords:** NAFLD, MAFLD, NASH, serum proteome, biomarker, cytokine signaling

## Abstract

**Background and aims:**

Non-alcoholic fatty liver disease (NAFLD) is the most common chronic liver disease. The prognosis may vary from simple steatosis to more severe outcomes such as nonalcoholic steatohepatitis (NASH), liver cirrhosis, and hepatocellular carcinoma. The understanding of the biological processes leading to NASH is limited and non-invasive diagnostic tools are lacking.

**Methods:**

The peripheral immunoproteome in biopsy-proven NAFL (n=35) and NASH patients (n=35) compared to matched, normal-weight healthy controls (n=15) was studied using a proximity extension assay, combined with spatial and single cell hepatic transcriptome analysis.

**Results:**

We identified 13 inflammatory serum proteins that, independent of comorbidities and fibrosis stage, distinguished NASH from NAFL. Analysis of co-expression patterns and biological networks further revealed NASH-specific biological perturbations indicative of temporal dysregulation of IL-4/-13, -10, -18, and non-canonical NF-kβ signaling. Of the identified inflammatory serum proteins, IL-18 and EN-RAGE as well as ST1A1 mapped to hepatic macrophages and periportal hepatocytes, respectively, at the single cell level. The signature of inflammatory serum proteins further permitted identification of biologically distinct subgroups of NASH patients.

**Conclusion:**

NASH patients have a distinct inflammatory serum protein signature, which can be mapped to the liver parenchyma, disease pathogenesis, and identifies subgroups of NASH patients with altered liver biology.

## Introduction

Non-alcoholic Fatty Liver Disease (NAFLD), the hepatic presentation of the metabolic syndrome, is the most common liver disease with a global prevalence estimated to 25% (1). Hepatic steatosis is a hallmark of this disease that includes a spectrum of pathological features ranging from benign steatosis to development of cirrhosis, causing a need for liver transplantation (2). Steatosis accompanied with inflammation is referred to as nonalcoholic steatohepatitis (NASH) (3). Patients with NASH are at a higher risk of developing hepatocellular carcinoma (HCC), even without underlying fibrosis (2). Hepatic inflammation is considered the main driving force behind fibrosis development and disease progression (2), but the nature of the inflammation is still largely unclear. Furthermore, it remains elusive why some patients develop more severe disease while others do not. Indeed, the interplay between NASH and a plethora of heterogeneous risk factors, a diverse clinical presentation as well as unpredictable outcomes suggests the existence of subgroups of NASH-patients with distinct pathophysiology (4). Understanding how inflammatory networks differ in NAFL and NASH would thus aid in our understanding of NASH pathogenesis. Several studies have assessed single cytokines in the serum of NASH patients, (e.g. CCL2, TNF, and CXCL10 (5)), as well as a range of other mediators such as microRNAs and genetic factors (6). However, while some reports have investigated multiple inflammatory mediators and identified several interesting targets (e.g., fibroblast growth factor (FGF-21), osteoprotegerin (OPG)) (7–9), we still lack a broader understanding of how circulating and intrahepatic inflammatory networks are altered in this disease.

Today, liver biopsy, an invasive procedure with considerable side-effects, is needed to determine if a NAFLD patient has NASH (10). This hamper both routine clinical management as well as the ongoing clinical trials evaluating novel NASH and/or fibrosis treatments. Previous efforts have commonly measured individual (or a few) inflammatory mediators in NASH-patients (5, 11–14), including cytokeratin (CK)-18, probably the most evaluated biomarker of NASH (15, 16). It has also been challenging to determine what constitutes a NASH-specific signature compared to what depends on underlying comorbidities such as obesity, hypertension, type 2 diabetes (T2D), or liver fibrosis, since all of these can contribute to NAFLD progression (17, 18).

In this work, we first performed a comprehensive screening for a large number of inflammatory serum proteins, using a commercially available sensitive proximity extension assay (PEA) technology, in a cohort of biopsy-verified NAFL and NASH-patients. Our aim was to identify a disease-specific inflammatory serum protein signature in NASH taking relevant comorbidities into account. We further explored the identified inflammatory NASH-signature by single cell and spatial hepatic transcriptomics to gain insight into NASH pathobiology. The results are discussed in relation to current knowledge of NASH biomarkers and in the context of NAFLD pathogenesis.

## Results

### NASH patients have a similar biochemical profile to NAFL patients except for signs of increased inflammation

**Figure 1:**
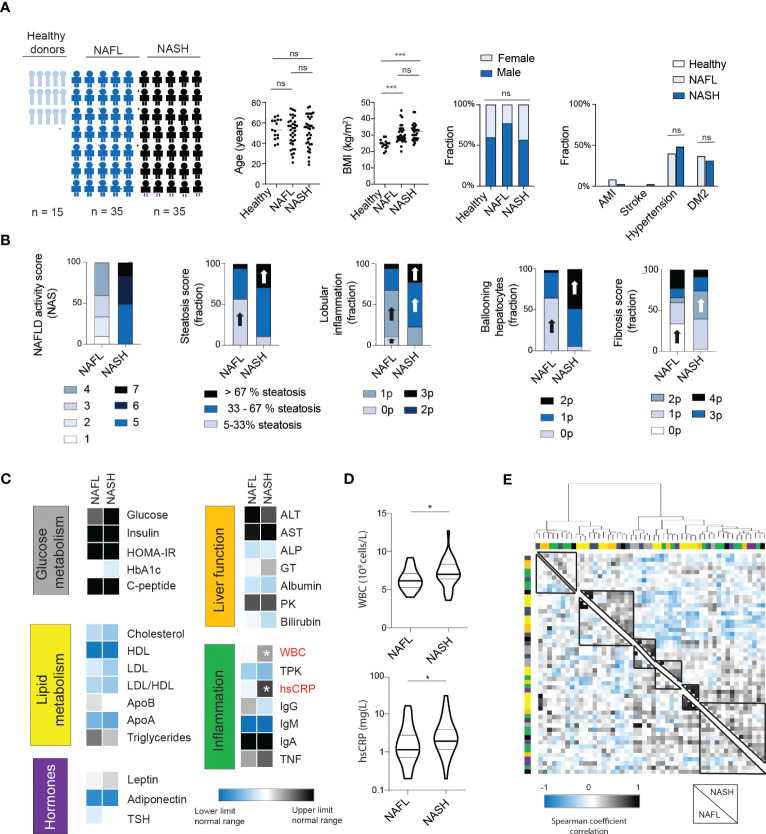
NASH patients have a similar biochemical profile to NAFL patients except for signs of increased inflammation. **(A)** Cohort characteristics including cohort size, age, BMI, sex as well as presence of comorbidities. Bars; median. **(B)** Stacked bar charts of histological features of NAFLD (n=35) and NASH patients (n=35), including NAFLD activity score (NAS), steatosis, lobular inflammation, ballooning hepatocytes (19) as well as fibrosis score (15). Block arrows; significantly larger fraction expression. **(C)** Heat map depicting median values of routine biochemical parameters for NAFL and NASH patients, in relation to the normal clinical range. **(D)** Violin plot of the levels of white blood cells (WBCs) as well as high-sensitive CRP (hsCRP) between the two groups, with median and quartiles indicated. **(E)** Heat map of hierarchical clustering of biochemical parameters. Cluster formation is based on the NASH-patients (upper right quadrant) or NAFL patients (lower left quadrant). Cluster analysis have been performed using Euclidian distances and Ward’s method. Color bars indicate the classification of the parameters (see **Figure S2** for details). Where applicable, *p-value of <0.05, ***p-value of <0.001, ns represents not significant. Parameters were analyzed using Mann-Whitney U-test (continuous) or Chi Square test (categorical).

To assess the inflammatory imprint of NASH on circulating proteins, serum was collected from 15 normal-weight healthy controls, 35 patients with NAFL, and 35 patients with NASH (**Figure 1A**). The cohorts were matched for age and sex. As expected, body mass index (BMI) was higher in patients compared to controls but not different between NAFL- and NASH-patients. Similarly, the prevalence of comorbidities associated with the metabolic syndrome, such as hypertension and type 2 diabetes (T2D), was present in similar levels in NAFL and NASH patients (**Figure 1A**). Instead, and expected, NASH-patients presented with a higher NAFLD activity score (NAS) and with increased levels of steatosis, lobular inflammation, hepatocyte ballooning, and liver fibrosis (**Figure 1B**). Next, in assessing 29 biochemical markers, covering glucose and lipid metabolism, hormones, liver function, as well as inflammation, only two inflammatory markers, total white blood cell count (WBC) and high sensitive C-reactive protein (CRP) were significantly elevated in NASH compared to NAFL patients (**Figures 1C, D** and **Figure S1**). Finally, to assess the systemic biochemical profile more globally in NAFL and NASH patients, we performed hierarchical clustering of the 29 biochemical markers analyzed (**Figure 1E**; **Figure S2**). While some parameters (e.g., those related to obesity) clustered together in both groups, there were also clusters of biochemical markers that differed between the two patient groups (**Figure 1E**; **Figure S2** **)**. Altogether, this suggests that NASH patients have a separate systemic imprint from NAFL and that the ongoing liver disease can in fact be detectable in the periphery. However, while the cluster analysis was able to distinguish differences between the pooled groups, it did not assist in separating individual NAFL from NASH patients based on routine biochemical tests.

### In-depth serum proteome analysis reveals an altered inflammatory profile in NASH-patients

**Figure 2:**
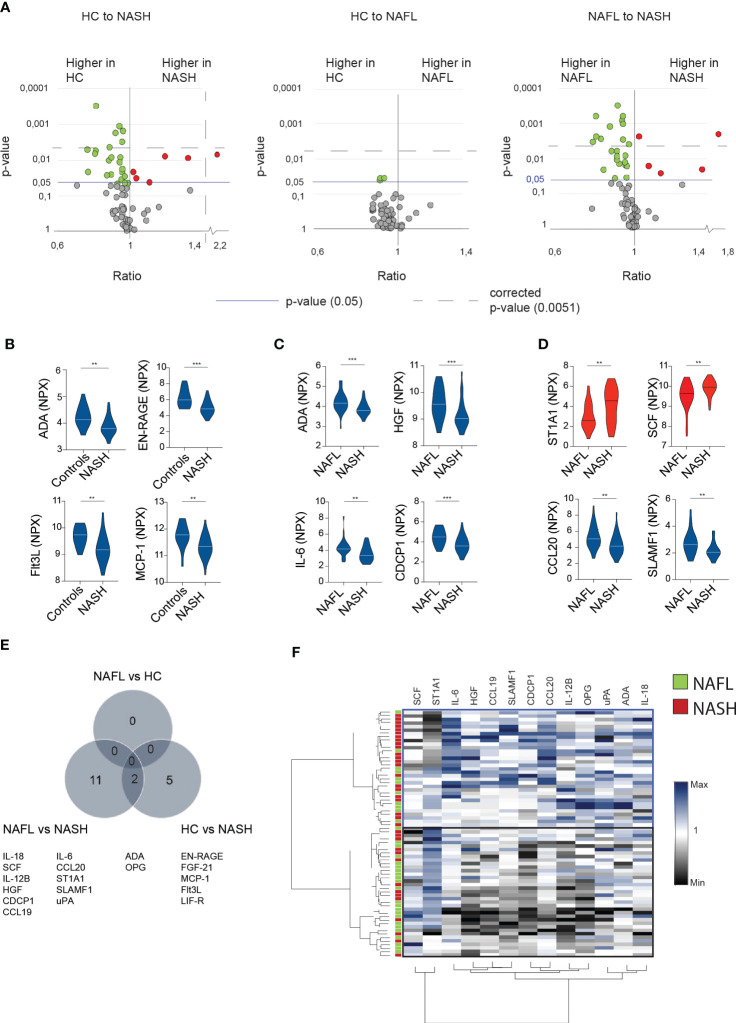
In-depth serum proteome analysis reveals an inflammatory serum protein signature in NASH-patients **(A)** Volcano plots depicting the ratio and p-values of measured cytokines in healthy controls (HC) compared to NASH-patients (left), healthy controls compared to NAFL patients (middle), and NAFL-patients compared to NASH-patients (right). Red dots; significantly elevated serum proteins. Green dots; significantly reduced serum proteins. Blue line; p-value of 0.05. Dotted line; the level of corrected p-values using the Benjamini-Yekutieli method of multiple correction with a Q of 5% (see Materials and methods for more details). **(B-D)** Violin plots showing examples of significantly altered serum proteins between the indicated groups. Elevated (red) or decreased (blue) serum proteins in NASH compared to other groups. **(E)** Venn-diagram of differentially expressed serum proteins **(F)** Hierarchically clustered heat map of the 13 significantly altered cytokines in NASH- compared to NAFL-patients. Presented values reflect the individual value of all included patients normalized by division with the median of the NAFL group. **p-value of < 0.01, ***p-value of <0.001. ADA, adenosine deaminase.

Since the main difference between the patient cohorts related to inflammatory markers, we next performed a deep serum proteome analysis of 92 inflammatory proteins (out of which 67 could be readily detected, see method section) using proximity extension assay (PEA) technology. This revealed that NASH-patients had a unique expression profile that was both distinct from NAFL-patients and healthy controls (**Figure 2A**). In contrast, no significant differences were observed when comparing NAFL-patients with healthy controls (**Figure 2A**). Surprisingly, most of the significantly altered inflammatory serum proteins were present at lower level in NASH compared to the other cohorts (**Figures 2B–D**; **Figure S3**) except for sulfotransferase 1A1 (ST1A1) and stem cell factor (SCF), that were both found at significantly higher levels in NASH (**Figure 2D**). While all the altered serum proteins were involved in inflammatory processes, few have previously been studied specifically in the context of NASH. Altogether, 13 inflammatory serum proteins were significantly altered in NASH compared to NAFLD and 7 when comparing NASH to healthy controls (**Figure 2E**; **Figures S3A–C**). To assess whether the 13 inflammatory serum proteins that differed comparing NAFL with NASH patients could be used to separate NASH from NAFL patients, we performed hierarchical clustering analysis (**Figure 2F**). While two major clusters were identified, these did not clearly separate the two cohorts (**Figure 2F**). However, since the inflammatory serum proteins also grouped into several clusters (**Figure 2F**), this suggested redundancy and indicated that more refined modelling of these altered factors might lead to a better separation of the two patient groups.

### The NASH serum signature is independent of specific comorbidities

**Figure 3:**
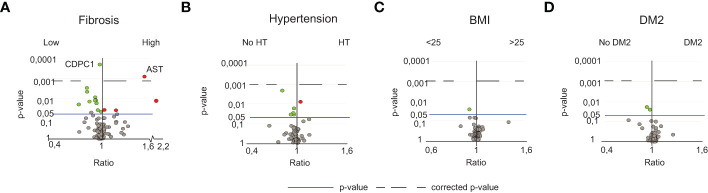
NASH-specific peripheral perturbations are independent of fibrosis, sex, age, BMI, hypertension and T2D. **(A)** Volcano plot depicting the ratio and p-values of the measured serum proteins in patients with low fibrosis (stage 0-1, n=35) or high fibrosis (stage 2-4, n=35). **(B)** Volcano plot depicting the ratio and p-values of the measured serum proteins in patients with (n=29) or without (n=31) hypertension, **(C)** individuals with normal (<25, n=15) or high BMI (>25, n=70), **(D)** individuals without (n=45) or with T2D (n=25). For details of diagnostic criteria regarding comorbidities, please see the methods section. Red dots; significantly elevated. Green dots; significantly reduced serum proteins. The line; p-value of 0.05. The dotted line; the level of corrected p-values using the Benjamini-Yekutieli method of multiple correction with a Q-value of 5%.

Next, we assessed whether the identified inflammatory serum protein signature was specific to NASH or associated with underlying comorbidities (fibrosis, T2D, hypertension, obesity) or other factors such as age and sex. When analyzing T2D, hypertension, obesity, age, and sex in relation to all serum proteins, no significant differences were observed (**Figures 3A–D**; **Figures S4A, B**). For fibrosis, when comparing patients with low vs significant fibrosis (stage 0-1 vs stage 2-4), while correcting for multiple comparisons, two serum proteins remained significantly different, aspartate aminotransferase (AST) and CDCP1 (**Figure 3A**; **Figures S4C, D**). A subgroup analysis revealed that AST indeed associated with higher levels of fibrosis, independent of NAFL or NASH whereas CDCP1 was rather dependent on NAFL or NASH (**Figures S4E, F**). This, together with the fact that CDCP1 showed no negative correlation to the degree of fibrosis but rather to NAFLD Activity Score (NAS) as a measurement of disease activity (data not shown), suggested that CDCP1 levels instead associated with active liver inflammation.

To further confirm that the identified inflammatory serum protein signature was independent of fibrosis we analyzed RNA-sequencing data of 34 NASH livers with low (n=15) or significant fibrosis (n=19) based on the expression of collagen transcripts, (**Figures S4G, H**). None of the serum proteins specifically changed in NASH (**Figure 2A**) were differentially expressed in NASH livers with low compared to significant fibrosis (data not shown). In contrast, this analysis identified genes known to be involved in the fibrotic process (*THBS2*), other genes previously identified in the context of hepatic manifestation of the metabolic syndrome or progressive fatty liver disease (*UBD, IL32*, *CD24*), as well as novel genes (*MIR4660, PHYH* and *PLA2G4C*) that might be involved in the fibrotic process specifically in NASH (**Figure S4H**).

In conclusion, the identified inflammatory serum protein signature in NASH was independent of fibrosis, hypertension, T2D, obesity, age, and sex.

### A combination of six inflammatory serum proteins segregates NASH from NAFL

**Figure 4:**
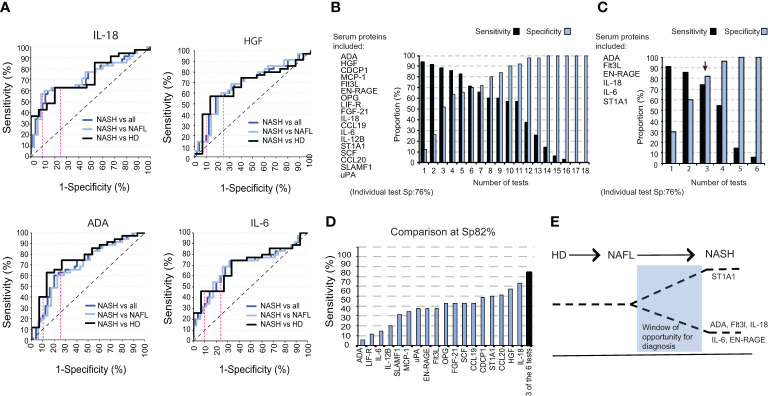
A combination of six inflammatory serum proteins segregates NASH from NAFL. **(A)** ROC curves for IL-18, HGF, ADA, and IL-6 for NASH versus NAFL and healthy controls combined, NASH versus NAFL-patients, and NASH versus healthy controls. **(B)** Bar graph of the sensitivity and specificity for a panel of tests including 18 significantly altered cytokines between NASH-patients and total non-NASH individuals. **(C)** Bar graph of sensitivity and specificity with a selected panel of six significantly altered serum proteins. **(D)** Bar graph of the sensitivity of each individual cytokine at a specificity of 82%, compared to the sensitivity of three positive tests from **(C)**. **(E)** Schematic model for a window in time when the identified signature could be used to separate NASH from NAFL.

We next evaluated the efficacy of each of the individual inflammatory serum proteins to differentiate NASH from NAFL and healthy individuals. To this end, we performed Receiver Operating Curve (ROC) analysis in which the individual proteins reached sensitivities ranging from 37% to 63% at a 76% specificity-level (Sp 76%)) and sensitivities from 9% to 43% at a 90% specificity-level (Sp 90%) (**Figure 4A**; **Figure S5A**). To improve this outcome, we next combined several proteins in the same analysis to increase both sensitivity and specificity. Using the previously determined thresholds of the individual tests (Sp 76% and 90%), and as an example, patients positive for 11 tests allowed a sensitivity of 57% at a specificity 92% (**Figure 4B**). As expected, this strategy also yielded a continuum of sensitivities (up to 94%) and specificities (up to 100%), from low to high depending on the number of tests but a gain in sensitivity was mirrored by a decrease in specificity and vice versa (**Figure 4B**; **Figure S5B**). This strategy also allowed for a significantly enhanced sensitivity at very high specificity-level (96%) as compared to using only a single protein in the analysis (**Figure S5C**). Since the hierarchical clustering of soluble factors significantly different in NASH compared to NAFL-patients suggested redundancy in how much a single protein contributes to separating the two groups (**Figure 2F**), we performed a logistic regression analysis. This identified ADA, FLt3L, EN-RAGE, IL-18, IL-6, and ST1A1 as the best combination of inflammatory serum proteins in segregating NASH from NAFL. Here, again, with a varying number of positive tests, a continuum of performances was evident (**Figure 4C**). However, a sensitivity of 74% at a 82% specificity-level could be achieved and this was higher than any single test (**Figures 4D, E**).

Overall, these results demonstrate the clinical potential of using the six identified inflammatory serum proteins in distinguishing NASH from NAFL.

### NASH associates with perturbations in co-expression patterns of inflammatory serum proteins

**Figure 5:**
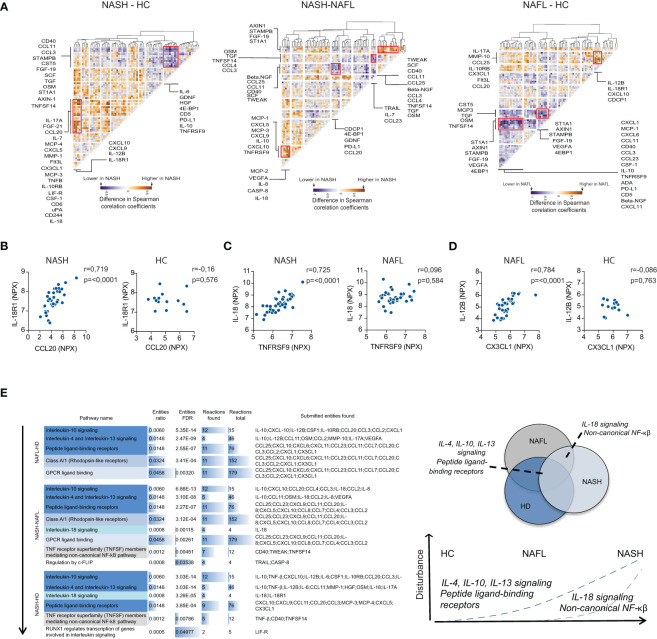
NASH associates with perturbations in co-expression patterns of serum cytokines. **(A)** Hierarchically clustered residual plots of correlation matrices of cytokine expression in NASH-, NAFL-patients, and healthy controls respectively. Clusters indicate differentially co-expressed cytokines between NASH-patients and healthy controls (HC) (left), NASH-patients and NAFL-patients (middle), and NAFL-patients and healthy controls (right). **(B–D)** Examples of differentially co-expressed serum proteins in **(B)** NASH-patients compared to healthy controls (HC), **(C)** NASH- compared to NAFL-patients, and **(D)** in NAFL-patients compared to healthy controls. R-values reflect the Spearman correlation coefficient and p-values as indicated. **(E)** Pathway analysis of differentially co-expressed cytokines between the three groups (left) and schematic model of disturbed pathways related to disease stages (right).

To gain more insights into NASH pathogenesis, we next set out to identify disease-specific signatures of serum proteins by analyzing the inter-dependency of the inflammatory serum proteome. First, we performed a correlation analysis of all measured serum proteins (**Figure S6A**). Based on this matrix we generated residual plots, comparing NASH to NAFL and healthy controls, that we finally performed hierarchical clustering on. This allowed us to identify clusters of deregulated inflammatory serum proteins specific to the different disease stages (**Figures 5A–D**; **Figure S6B**). We found four distinct clusters of deregulated cytokines that differed between NAFL and NASH patients as well as two clusters that separated NASH-patients from healthy controls. Of note, by assessing the association between inflammatory serum proteins, disturbances also emerged between NAFL patients and healthy controls that were not detected when only the expression patterns of single proteins were considered. Finally, we performed pathway analysis based on inflammatory serum proteins from the identified dysregulated clusters (**Figure 5E**). The pathway analysis indicated a temporal deregulation of type 2 immunity (IL-4, IL-13 axis) as well as IL-10, IL-18, and non-canonical NF- κβ signaling in NASH, where dysregulation of IL-18 and non-canonical NF-κβ signaling was specific for NASH patients (**Figure 5E**). In summary, we here demonstrate that perturbations in systemic co-expression patterns of inflammatory serum proteins reveal a map of distinct signaling pathways in NAFL and NASH.

### Mapping the NASH inflammatory serum protein signature onto the liver parenchyma at the single-cell level

**Figure 6:**
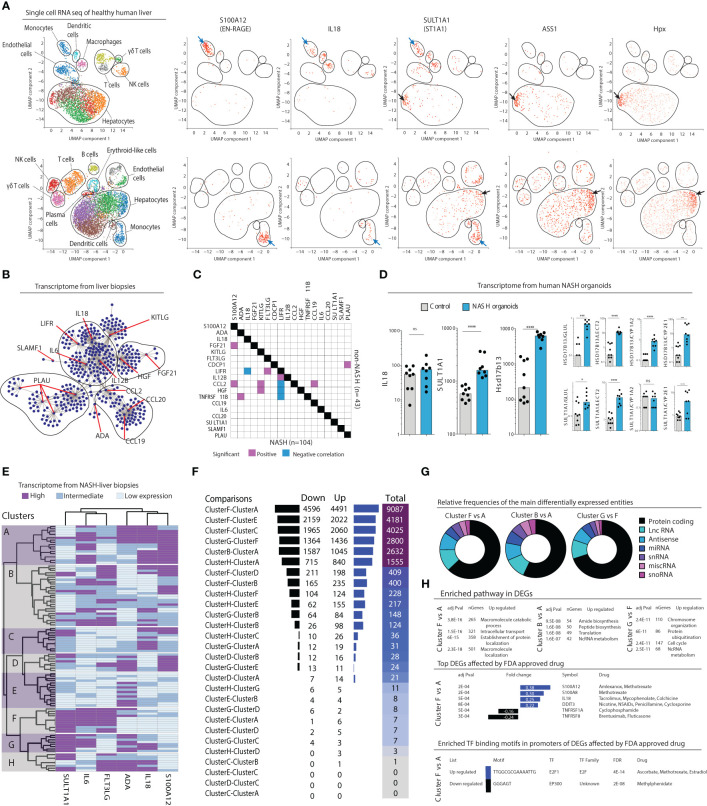
Inflammatory serum protein signature identifies distinct subgroups of NASH patients. **(A)** Visualized cell clusters from single-cell RNA-sequencing of healthy human liver (n=2) as well as the individual expression of the genes *S100A12*, *IL18*, *SULT1A1*, and the hepatocyte-related genes *ASS1* and *Hpx*. **(B)** Biological network analysis of transcriptomes from liver biopsies (n=152). **(C)** Correlation matrix of the expression of 18 deregulated inflammatory serum proteins in the transcriptomes in NASH (n=104) compared to non-NASH livers (n=43). **(D)** Transcript levels of the genes *IL18*, *SULT1A1*, and the periportal marker *Hsd17b14* in human primary organoids of NASH-development, compared to control organoids. *p-value < 0.05, ***p-value < 0.001, ****p-value < 0.00001, ns, not significant. **(E)** Hierarchical clustering based on expression of the indicated six genes based on the transcriptome of 104 liver biopsies from patients with NASH. **(F)** Differentially expressed genes between combinations of the 8 clusters identified in **(E)**. **(G)** Pie charts of the relative frequencies of the main differentially expressed entities between the identified clusters. **(H)** Gene enrichment analysis of the differentially expressed genes (bottom).

Given the constant bidirectional exchange between liver and circulation, we hypothesized that the inflammatory serum protein signature also could be mapped to the liver parenchyma and distinct subsets of intrahepatic cells. To explore what cell types could be linked to the serum protein signature, we first analyzed publicly available single cell RNAseq data from two human healthy livers (**Figure 6A**). This analysis revealed that a specific population of myeloid cells expressed *S100A12* (EN-RAGE), *IL18*, and *SULT1A1* (ST1A1*)* transcripts. This population co-expressed *S100A8, S100A9, VCAN*, and *LYZ*, but not *CD5L, MARCO*, and *VCAM1* (**Figure S7A**) therefore corresponding to the recently described inflammatory macrophages (20) and/or tissue monocytes (21). In addition, *SULT1A1* (ST1A1*)* transcripts were detected in a subset of hepatocytes exhibiting a periportal signature with co-expression of *ASS1, Alb, Sds, Hsd17b13*, and *CPS1* (**Figure 6A**; **Figure S7B**), but none of the pericentral hepatocyte markers *Glul, Gulo, Lect2*, or *Gstm3* (data not shown). Thus, these results associate a periportal subset of hepatocyte as well as inflammatory tissue macrophages/monocytes to the NASH-specific inflammatory serum protein signature.

### Disturbances in inflammatory serum proteins linked to intrahepatic NASH pathogenesis

After having concluded that many of the inflammatory serum proteins that made up the NASH-signature were expressed in the liver we next set out to investigate their association more specifically to NASH in the liver. First, we performed a biological network analysis using RNAseq data from 152 liver biopsies of patients with suspected NAFLD and/or candidates for bariatric surgery, from a public available data repository (*GSE83452* (18)). This revealed that a majority of the 17 identified inflammatory serum proteins that were differentially regulated in NASH (**Figure 2**) indeed have known connections in the liver suggesting that they might be part of a biological network deregulated in NASH (**Figure 6B**). To evaluate if such a network was perturbed in NASH livers, we analyzed the liver transcriptome according to presence of NASH (n=104) or non-NASH (n=43) (*GSE83452* (18)). Correlation analysis showed that several of the identified inflammatory serum proteins were transcriptionally associated in the liver of NASH-patients but not in non-NASH livers (**Figure 6C**). Since biological sampling of NASH patients often occur late in the disease process, we also turned to human primary liver organoids to gain insight into the early dynamics of NASH pathogenesis. In this system, organoids, made up of primary liver cells such as hepatocytes, hepatic stellate cells, and macrophages, are exposed to a lipotoxic milieu over a period of ten days (20). As compared to control organoids, we observed that transcript levels of *IL18* were not altered in NASH organoids, which is in line with the results from the pathway analysis that suggest a later dysregulation of IL-18 signaling (**Figure 5E**). Instead, *SULT1A1* (ST1A1) as well as the periportal marker *Hsd17b13* were significantly upregulated in NASH organoids (**Figure 6D**). These results remained significant after correction for multiple pericentral hepatocyte markers indicating a perturbation of the pericentral/periportal hepatocyte transcriptional program (**Figure 6D**).

Taken together, this show that the inflammatory serum protein signature of NASH can be linked to disease pathogenesis in the liver and that deregulation in hepatocytes (ST1A1) might occur before appearance of the broader NASH-specific inflammatory signature.

### The inflammatory serum protein signature identifies distinct subgroups of NASH-patients

Finally, we asked whether the six identified inflammatory serum proteins that were superior in separating NASH from NAFL (**Figure 4C**) could be used to understand NASH heterogeneity. Using RNA sequencing data from 104 NASH livers from the previously mentioned data set (*GSE83452* (18)), we performed a hierarchical clustering analysis based on expression of these six serum proteins. This analysis identified eight distinct clusters of patients (**Figure 6E**). In performing pair-wise analysis of differentially expressed genes (DEGs) in-between the clusters, we noted that many of them had a similar liver transcriptome but that some clusters, such as A, B, F, and G, differed considerably in their transcriptomes (**Figure 6F**; **Figure S8A**). Of note, a main difference between clusters A and B compared to F and G was high vs low *SULT1A1* (ST1A1) expression. As a control, when only stratifying patients based on high vs low *SULT1A1* (ST1A1) expression, few, if any, significant differences in gene expression were noted (**Figures S8A, B**) suggesting that the refined classification of patients based on the six serum proteins specifically allowed the identification of these subgroups of patients. Furthermore, when modelling random clusters of patients (same size as those identified by the six inflammatory serum cytokines) and comparing them against each other, few DEGs were found (**Figure S8D**), suggesting biological relevance of clusters and DEGs identified based on the inflammatory serum cytokine signature. Assessing DEGs among the most differing NASH-patient clusters revealed that two-thirds of all genes were located to the protein coding compartment (**Figure 6G**). Moreover, this analysis identified specific biological pathways as well as tentative druggable genes and transcription factor binding motifs that were unique to each of the most distinct clusters (**Figure 6H**).

In summary, the inflammatory serum protein signature identified in NASH is not only coupled to the disease pathogenesis within the liver but also suggests a heterogeneous pathophysiology in NASH with distinct subgroups of patients.

## Discussion

NAFLD is becoming an increasing health problem worldwide due to severe long-term outcomes such as liver failure and HCC. Yet, challenges remain in understanding the disease pathophysiology to allow for novel therapies to be developed and with respect to optimal diagnosis and monitoring of NASH patients. Indeed, NASH is a major outcome in many ongoing pharmacological studies, however NASH is ill-defined and suffers from sampling errors. An objective marker for NASH would therefore be important. Our work presents a broad assessment of the peripheral proteome in NASH-patients from which we identified a NASH-specific inflammatory serum protein signature that could aid in separating NASH- from NAFL-patients. It further pinpointed several cytokine-signaling pathways that were altered throughout the NAFLD disease process. Finally, by overlaying the NASH-signature onto liver transcriptomes of a large group of NASH patients, we uncovered several clusters of patients with distinct liver biology. Thus, by performing an in-depth assessment of the peripheral immunoproteome, we here provide new insights into NASH pathophysiology.

Intriguingly, our study reports decreased levels of several pro-inflammatory cytokines in NASH that have been associated with the metabolic syndrome, such as IL-6 and IL-18. While increased levels of IL-18 have been observed in several models of obesity and the metabolic syndrome (22–24), the data on IL-18 in NAFLD have been far more ambiguous. In mice, IL-18 deficient mice develop hyperlipidemia and subsequent NASH (25, 26). Other studies have demonstrated increased levels of intrahepatic IL-18 in mice and humans (27, 28). However, data from patients with NAFLD is scarce and previous studies have not always differentiated between NAFLD or NASH-patients, nor taken the presence of obesity into account as a possible confounding factor (29, 30). However, the few studies that have previously assessed circulating levels of IL-18 in NAFL compared to NASH patients and taken the metabolic status of patients into consideration have not been able to demonstrate any differences (30, 31). It is plausible that our use of the novel PEA technology, which is more sensitive compared to standard enzyme linked immunosorbent assay (ELISA) (32, 33), combined with our sizeable and controlled cohort allowed us to demonstrate a previously unappreciated difference in IL-18 levels in NASH-patients. Interestingly, this is in line with results in murine models that highlights the importance of IL-18 in negatively regulating NAFLD/NASH development through inflammasome activation mediated by gut microbiota (34). Similar to IL-18, pro-inflammatory IL-6 has previously been linked to NAFLD development (13, 35, 36). While some studies display increased IL-6 in NASH compared to NAFL-patients (11), others report results in line with our findings with a tendency towards lower levels in NASH (35). Our results, suggesting heterogeneity in the IL-6 expression, as well as the strong association between IL-6 and visceral adiposity (37, 38), could provide an explanation to these conflicting results.

Our results highlight six inflammatory serum proteins that differ between NAFL and NASH-patients (ST1A1, ADA, Flt3L, EN-RAGE, IL-6, IL-18). Human sulfotransferase 1A1 (ST1A1) is an important enzyme in several biological process (sulfate conjugation of neurotransmitters, hormones, drugs and xenobiotics) (39). While found highly enriched in liver tissue, ST1A1 has not been described in NAFLD/NASH nor other liver diseases previously. However, inflammatory factors can increase the activity of ST1A1, which together with the increased levels in NASH suggest that it would be an interesting target for monitoring inflammatory processes. Adenosine deaminase (ADA) is a key enzyme in purine metabolism and is important for the maintenance of the immune system. Decreases in ADA might result in dysfunction within cellular immunity (40), which is a known component of NASH. It can also reduce the levels of adenosine and increase glycolysis (41). The serum activity of one of the two ADA isoforms (ADA2) has recently been associated to fibrosis development in NAFLD patients, possibly due to a shift in macrophage polarization towards profibrotic, type 2 macrophages (42). While our results demonstrate lower levels of total ADA in NASH-patients compared to NAFL, probably highlighting important roles besides its role in fibrosis development, this emphasizes ADA as an interesting molecule for NAFLD development and emphasizes the need of future studies to fully elucidate its role. FMS-like tyrosine kinase 3 ligand (Flt3L) is a cytokine and growth factor that is important for replenishment and homeostasis of immune cells as well as regulating DC function, also in the context of ameliorating liver injury (43). Flt3L also positively correlate to the levels of myocardial fat deposition in NAFLD patients (44). However, to the best of our knowledge, there are no previous reports on the role of Flt3L in NASH. Our results also show a decrease in EN-RAGE (also known as S100 calcium-binding protein A12, S100A12). Metabolic dysfunction increases the formation of advanced glycation end-products (AGEs), which can contribute to liver damage (45). Soluble receptors (sRAGEs) can act as decoy receptors thus preventing activation of the AGE/RAGE axis that contributes to inflammation (46). sRAGE negatively correlate with ALT in NAFLD patients and lower levels of sRAGEs are present in individuals with metabolic disease (46). Taken together, our identified inflammatory serum protein signature novel proteins that have previously not been specially studied in a NAFL/NASH context but that have functions linked to metabolism and inflammation suggesting relevant roles for them in NASH. However, how they compare to other biomarkers of NASH remains to be further studied.

NAFLD is multifactorial disease, thought to be driven by a multitude of parallel processes such as hepatocyte death, wound healing, continuous fibrogenesis/fibrinolysis, angiogenesis, as well as inflammation (47). One aspect of the lipotoxicity that affects NASH-livers is the capacity to interact with and modify signaling pathways that regulates metabolism, stress, and inflammatory responses (47). Our pathway analysis revealed several disturbed signaling pathways during NAFLD development. One highlighted target was NF-κβ signaling, that regulates the expression of several pro-inflammatory cytokines, including IL-6. During insulin resistance, a common feature of NASH, the expression of NF-κβ in the liver is increased and NF-κβ is closely linked to hepatic insulin resistance (45). Thus, our results on disturbances in NF-κβ signaling are in line with previous hypothesis on how NF-κB is involved in the pathogenesis of insulin resistance and NASH, thus again highlighting the importance of NF-κB signaling in NASH.

NAFLD is a heterogeneous disease with a fluctuating disease progression. The degree of steatosis as well as liver inflammation can show a dynamic progression, shifting between NAFL and NASH over time, which is difficult to assess using infrequent biopsies. However, fibrosis deposition remains a more stable measurement than the hepatic inflammation (2). Thus, it is likely that the patients are sampled at different time-points since the disease debut and in different stages of the disease. Despite this, our identified NASH-signature was independent of the stage of fibrosis. This is a strength, since there is a lack of non-invasive tools to measure the more rapidly fluctuating hepatic inflammation, in contrast to several existing strategies to measure intrahepatic fibrosis. When new treatments have been tested on NASH patients, considerable heterogeneity in response rates have been noted (48). This raises the possibility that subgroups of patients might exist with distinct underlying biology. Indeed, by clustering whole liver transcriptome data from a large cohort of NASH-patients based on expression of the six identified inflammatory serum proteins, we identified subclusters of patients showing distinct liver biology as well as different imputed responses to treatments. It is plausible that future clinical trial design in NASH could be informed by knowledge about such patient heterogeneity.

Certain limitations of our study should be considered. First, the usage of a pre-selected panel of inflammatory serum proteins instead of more unbiased approaches such as proteomics. However, this was within the aim of the design to in depth study the inflammation in NASH and in large focus on the inflammatory component of NASH. Of course, other pathophysiological processes are important in NAFLD, e.g. fibrosis development, angiogenesis, oxidative stress. Second, how our proposed serum protein signature compare to other NASH biomarkers needs to be further assessed in future studies. Third, our cohorts were relatively small and the study design cross-sectional. While the study design provides an interesting correlation between histological features and serum proteins, how, and if these serum proteins correlate to clinical outcomes remain to be further studied. Forth, large multicenter clinical trials would be able to investigate how the identified heterogeneity of NASH detected in our study would relate to clinical outcomes as well as treatment responses.

## Conclusions

We have performed a large and detailed analysis of serum proteome changes in NASH patients. In addition to identifying an inflammatory serum protein signature that was specific to NASH and not associated with liver fibrosis or other comorbidities, we also associate this to specific and aberrant cytokine signaling pathways in NASH-patients. Finally, we highlight the role of hepatic macrophages and periportal hepatocytes in the pathogenesis of NASH and identify subpopulations of NASH-patients with distinct liver biology which could help to understand the heterogeneity of the disease.

## Materials and methods

### Study design and cohort characteristics

The study was approved by the regional ethics committee in Stockholm (Dnr: 2013/2285-31/3, Dnr: 2006-229-31-3) and oral and written informed consent was obtained from all participants. Serum from 15 healthy controls was collected from Skanstull Blood Donor Center, Stockholm. Exclusion criteria were presence of type 2 diabetes, BMI>25, or elevated ALT. Serum as well as clinical data from patients were collected at routine visits at the out-patient clinic at the Division of Hepatology, Department of Upper GI, Karolinska University Hospital, Stockholm, Sweden. All patients had biopsy-verified NAFL (n=35) or NASH (n=35), no other liver diseases nor a weekly alcohol consumption of less than 30g for men and 20g for women. In addition, information on comorbidities was collected. Type 2 diabetes (T2D) was defined as a registered diagnosis in patient charts, a non-fasting glucose value of ≥180 mg/dl or a fasting glucose value of ≥126 mg/dl, or having any anti-diabetic medication prescribed. Hypertension was defined as a registered diagnosis in patient charts, a resting blood pressure of ≥140/90 mmHg, or having any anti-hypertensive medication prescribed (49).

### Sensitive quantification of serum proteins by proximity extension assay

Serum protein data was generated using the commercial proximity extension assay technology (PEA, ProSeek, Olink AB, Uppsala). In more detail, 25μl serum per patient was aliquoted into a 96-well V-plate and frozen down at -80C until analysis. Next, 92 pre-selected cytokines from a commercial platform (Inflammation panel from Olink Proteomics) were analyzed using PEA. The specificity and sensitivity of the assay as well as the method for normalization and quantification of the data was at length described by Assarsson et al (50). In short, serum proteins were detected by paired oligonucleotide-coupled antibodies, which when in proximity after binding to a serum protein becomes the target of a polymerase that creates unique sequences. These unique surrogate markers can then be quantified using real-time PCR (50). One advantage of the method is increased sensitivity compared to multiplex immunoassays and due to the detection of only matched DNA pairs the risk of antibody cross-reactivity, as seen e.g. in ELISA multiplex assays, is lower. The assay utilizes internal controls to assess interplate variation as well as being utilized for normalization. Out of 92 serum cytokines tested for, 67 were detected in the analyzed samples, due to technical reasons. An additional four cytokines were excluded from downstream analysis due to more than 10% of samples being below the cutoff limit of minimum detection. Thus, only serum proteins that were detected in >90% of the samples were analyzed. Data is presented as normalized protein expression (NPX), which has been corrected for technical variation.

### Assessment of peripheral biochemical parameters

Several routine biochemical variables were collected at the same day as the serum sample. Standard measurements of liver function were assessed, including levels of alanine (ALT), aspartate aminotransferase (AST), and bilirubin. All patients were negative on hepatitis virus serology. Glucose metabolism was evaluated using fasting glucose, insulin, and HbA1c. Fasting glucose was measured after 6h of fasting and HOMA-IR was calculated. Lipid metabolism assessment included triglycerides, LDL, HLD, and apolipoproteins A. In addition, the height and weight of the patients, including hip and waist circumference, was measured by a nurse and used for calculation of BMI (kg/m^2^). P-Ethanol and CDT was measured and used as an objective evaluation of excessive alcohol use.

### Histological evaluation of liver biopsies

All biopsies were scored by an experienced liver pathologist according to NAFLD activity score (NAS) (where NASH was defined as a NAS ≥5) (19). Steatosis was graded according to the frequency of hepatocytes with cytoplasmic lipid accumulation, where 0 points (0) represents <5% steatosis which is incompatible with a NAFLD diagnosis (5%, 0p; 5%-33%, 1p; 33%-66%, 2p; 66%, 3p). Lobular inflammation was scored with 0-3 points according to number of inflammatory foci/200x field (no inflammatory foci, 0p; 2 foci/200 field, 1p; 2-4 foci/200 field, 2p; >4 foci/200 field, 3p). Ballooning was also measured according to NAS on a 2-point scale where 0p represents no ballooning hepatocytes, 1p represents few ballooning hepatocytes and 2 p represents many cells. Fibrosis stage was scored according to the Kleiner classification on a 5-point scale (F0–F4) (19, 51).

### Analysis of publicly available transcriptomic datasets

Several publicly available data repositories were used to validate our results. Detailed methods descriptions for these datasets can be found in the respective publications as listed below. In **Figure 6A**, publicly available single cell RNAseq data from two human healthy livers, sequenced in order to perform a comprehensive atlas of immune cells were used to map our serum proteins to cells of origin in the liver (20). The cells were derived from human donor livers that were considered healthy enough to undergo liver transplantation. The single cell RNA sequencing analysis was performed using Panglao DB (6806 cells and 4190 cells generated on 10X chromium platform (GSM3178786, GSM3178782 (20)). The raw data can be found at he Gene Expression Omnibus at the National Institute of Health (https://www.ncbi.nlm.nih.gov/geo/). Publicly available transcriptome data from NASH livers was used to analyze the transcripts of our protiens of interest in **Figure 6**. The data was generated from 152 patients who underwent gastric bypass surgeries. After histological assessment, using the NASH Clinical Research Network (NASH CRN) Scoring System, where the presence of NASH was defined according to Chalasani et al. necessitating the combined presence of steatosis, ballooning and lobular inflammation, they were divided into 44 non- NASH, 104 NASH, and 4 undefined (52). The transcriptome was obtained by Affymetrix Human Gene 2.0ST array (GSE83452 (52)). To further validate our results in an early model of NASH we utilized publicly available data on liver organoids (**Figure 6D**). In short, the organoids were created by *in vitro* co-culure of primary hepatocytes, hepatic stellate cells and macrophages, which were exposed to lipotoxic stress factors (glucose, insulin and free fatty acids) (53). The trancriptomic data was generated by Illumina HiSeq 2000 platform (GSE89063 (53))

### Strategy for data and statistical analysis

Differences between groups were assessed by Mann-Whitney or Chi Square tests where α<0.05 was considered as significant. Correction for multiple comparisons was performed using two-stage linear step-up procedure of Benjamini, Kriger and Yekutieli and Q-value threshold for significance was set at 5%. Coefficients of correlation were computed using the Spearman rank method and residual plots were generated by substraction of these coefficients. Paired proportions for diagnosis tests were compared using Mc Nemar test. These analyses were performed using Prism Version 8.3 (GraphPad Software Inc; USA). Hierarchical clustering (using Euclidian distances and ward method) and logistic regression were computed in R (R Core Team). Cluster analysis have been performed using Euclidian distances and Ward’s method due to the absence of arbitrary seeding, the maximized of inter-cluster variance, and the minimized intra-cluster variance. Pathway analysis was performed using the Reactome (54). Network analysis was performed using Cytoscape (3.7.2). The differential gene expression analysis was perform using iDEP (55), and drug gene interactions were identified using the drug gene interaction database (56).

## Data availability statement

The original contributions presented in the study are included in the article/**Supplementary Material**, further inquiries can be directed to the corresponding authors.

## Ethics statement

The studies involving human participants were reviewed and approved by the regional ethics committee in Stockholm (Dnr: 2013/2285-31/3, Dnr: 2006-229-31-3). The patients/participants provided their written informed consent to participate in this study.

## Author contributions

Conceptualization, NS, MC, and NB. Methodology, NS, MC, and NB. Formal analysis, NS, MC, and NB. Investigation NS, MC, and NB. Resources, HH and PS. Writing – Original draft, NS, MC, and NB. Writing – Review and Editing, all authors. Visualization, NS, MC, and NB. Supervision, MC, and NB. Funding acquisition, NB. All authors contributed to the article and approved the submitted version.
