# Supplementary material for: Dysregulated peripheral proteome reveals NASH-specific signatures identifying patient subgroups with distinct liver biology
Source: Front Immunol. 2023 Jun 5;14:1186097. doi: 10.3389/fimmu.2023.1186097 (PMC10277514; doi:10.3389/fimmu.2023.1186097)
Supplement: Supplementary file 1 [file DataSheet_1.pdf]

Supplementary figures

Supplementary figure 1. Soluble factors related to liver function, lipid metabolism, hormones, glucose metabolism, and inflammation in NAFLD.

Scatter plots of the levels of biochemical parameters in NAFL patients compared to NASH-patients. Red bars reflect median. Indicated lower (blue) and upper levels (black) of normal range as determined by the local laboratory routines. Ns = not significant.

Supplementary figure 2. Hierarchical clustering of soluble biochemical parameters from NAFL patients identifies NASH-specific clusters of correlation. The top square reflects the correlation matrix of the measured parameters in NASH-patients (n=35) with 6 identified clusters. The bottom square reflects the correlation matrix of the same parameters in NAFL-patients (n=35). Colors represent the classification of the parameter into five different categories: green – inflammation, orange - liver function, dark grey – electrolytes, light gray – glucose metabolism, and purple – hormones. Spearman correlation coefficients are shown according to the indicated scale.

Supplementary figure 3. NASH patients have significantly altered inflammatory serum proteins compared to NAFL patients and healthy controls.

(A) Scatter plots of the 7 significantly differentially expressed serum proteins between NASH-patients (n=35) and healthy controls (n=15).

(B) Scatter plots of the 13 significantly differentially expressed serum proteins between NASH-patients (n=35) and NAFL-patients (n=35). Red line represents median values. (C) Venn diagram of the number of overlapping significantly altered serum proteins, before adjusting for multiple comparisons.

Supplementary figure 4. The NASH-imprint of inflammatory serum proteins is independent of fibrosis.

(A) Volcano plots of the levels of inflammatory serum proteins in patients < 50 years (n=30) or > 50 years of age (n=40).

(B Volcano plots of the levels of inflammatory serum proteins in women versus men.

(C) Scatter plot of aspartate aminotransferase (AST) in patients with low or high levels of fibrosis.

(D) Scatter plot of CDCP1 in patients with low or high levels of fibrosis.

(E) Scatter plot of aspartate aminotransferase (AST) in patients with low or high levels of fibrosis, divided according to presence of NAFL or NASH.

(F) Scatter plot of CDCP1 in patients with low or high levels of fibrosis, divided according to presence of NAFL or NASH.

(G) Levels of gene transcripts of three different collagens from 34 livers (low fibrosis n= 15, high fibrosis n=19).

(H) Differentially expressed genes between livers with high compared to low levels of liver fibrosis identified as in (E). Black bar represent median. * p-value of <0.05, ** p-value of < 0.01, *** p-value of <0.001.

Supplementary figure 4. Sensitivity and specificity of all significantly altered serum proteins in distinguishing NASH from NAFL.

(A) ROC curves for the 13 significantly altered serum proteins in NASH-patients compared to NAFL-patients.

(B) Bar graph of sensitivity and specificity of combinations of measured serum proteins.

(C) Bar graph depicting the sensitivity of combinations of tests compared to the indicated individual tests.

Supplementary figure 5. Analysis of correlation patterns reveal deregulated covariation of inflammatory serum proteins.

(A) Hierarchically clustered correlation matrix of all 67 measured inflammatory serum proteins in NASH-, NAFL-patients, or healthy controls (HC) respectively. Values reflect Spearman correlation coefficient.

(B) Hierarchically clustered residual plots of correlation matrices of cytokine expression in NASH-, NAFL-patients, and healthy controls respectively.

(C) The top-ten most deregulated co-expressions of inflammatory serum proteins in NASH compared to healthy controls, NASH compared to NAFL, and NAFL compared to healthy controls, respectively. R represent Spearman correlation coefficient.

Supplementary figure 7. In-depth analysis of gene expression in myeloid cells as well as hepatocytes from healthy livers.

(A) Gene expression of *S100A8, S100A9, VCAN, LYZ*, *CD5L, MARCO*, and *VCAM1* in intrahepatic myeloid cells from scRNA data from two healthy donor livers.

(B) (top) Gene expression of the genes *albumin*, *sds*, *Hsd17b13,* and *csp1* (bottom) reveals a periportal hepatocyte signature.

Supplementary figure 8. Gene expression in clusters of NASH-patients.

(A) Volcano plots of differentially expressed genes between cluster A and cluster H.

(B) Volcano plot of differentially expressed genes in livers with high versus low levels of *SULT1A1*.

(C) Differentially expressed genes between cluster A and F, A and B, as well as cluster G and F.

(D) Table presenting differentially expressed genes (DEGs) from gene analysis of 10 repeated randomizations of all patients.

Supplementary figure 1

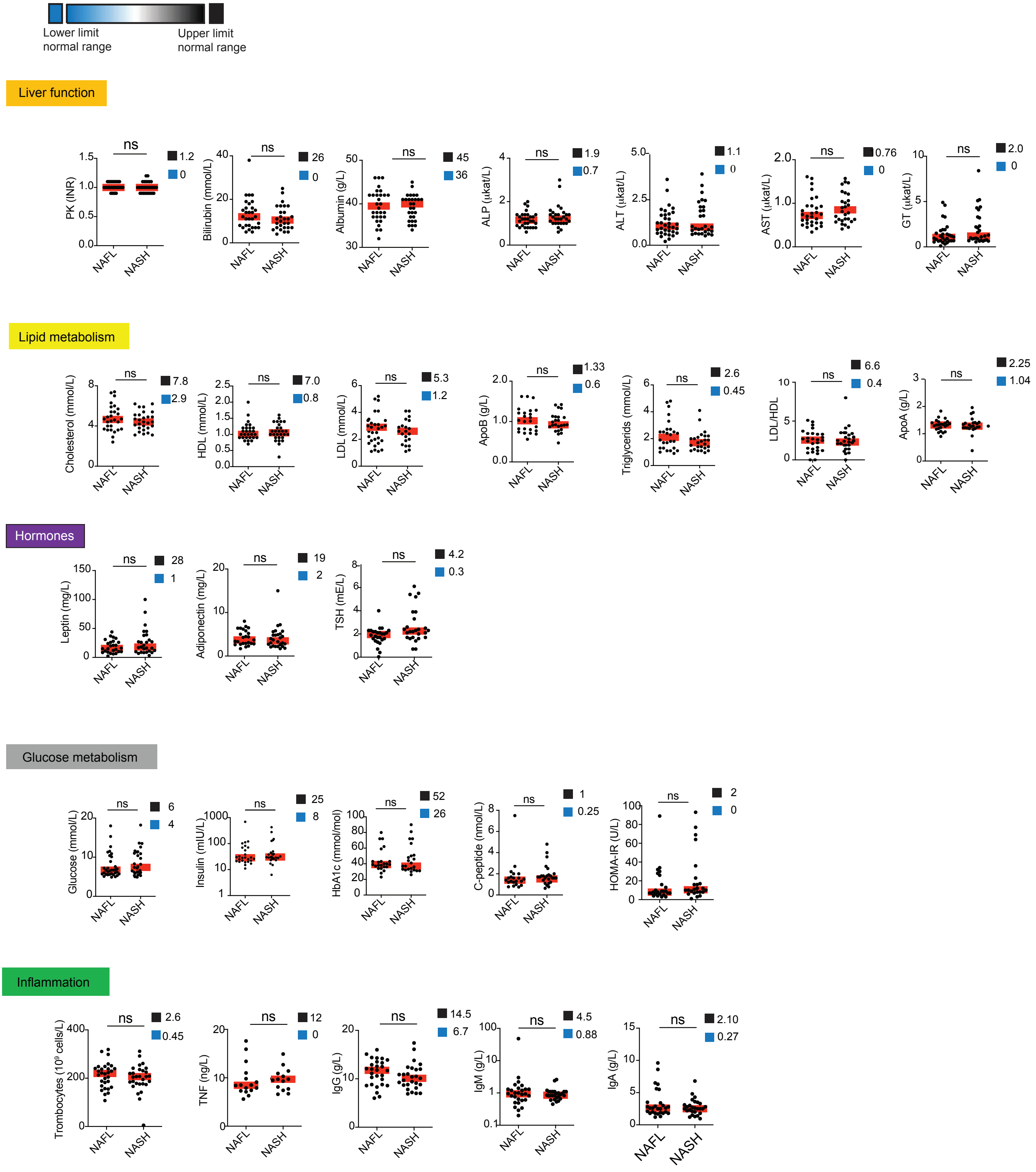

Supplementary figure 2

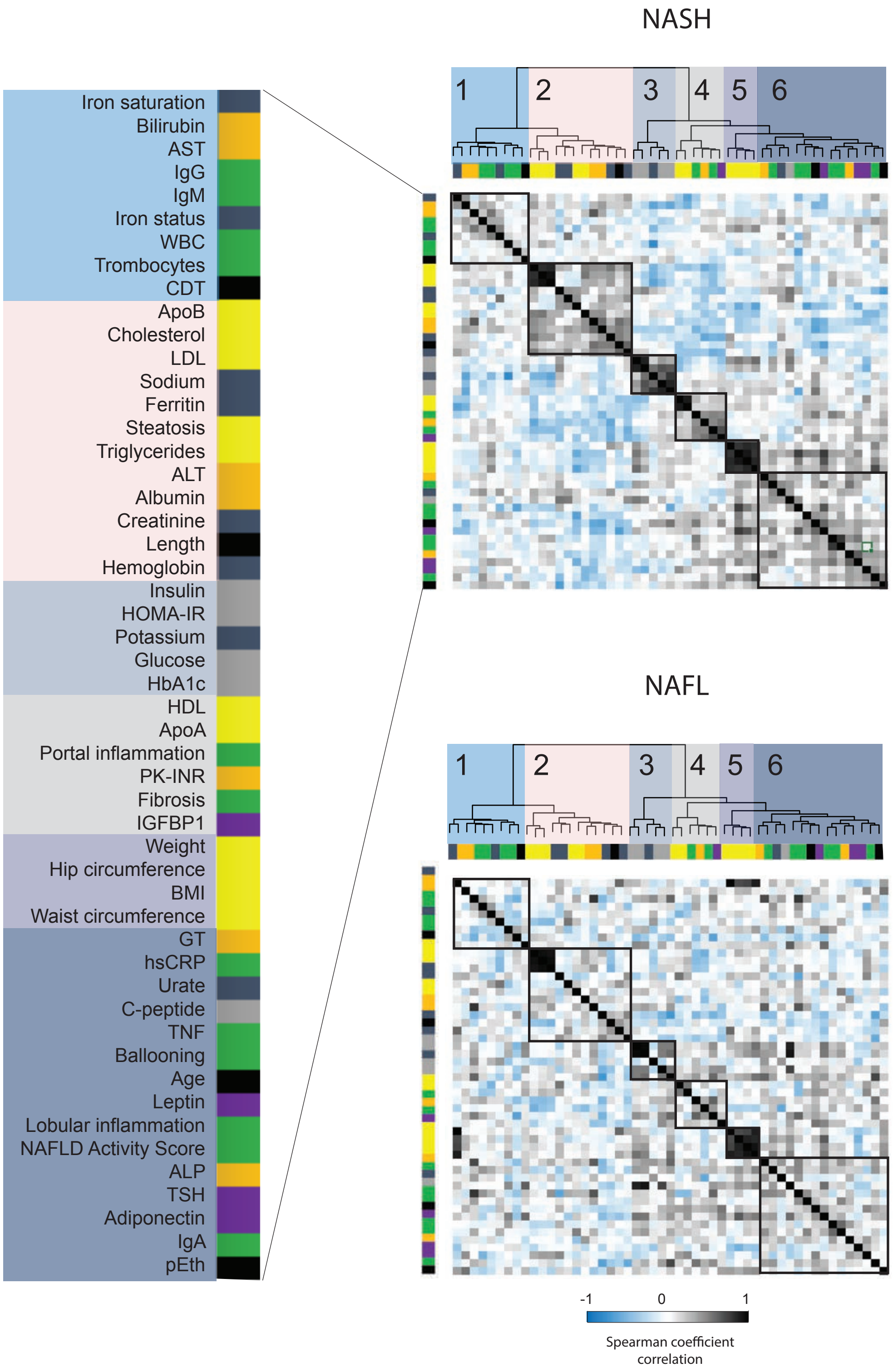

Supplementary figure 3

A

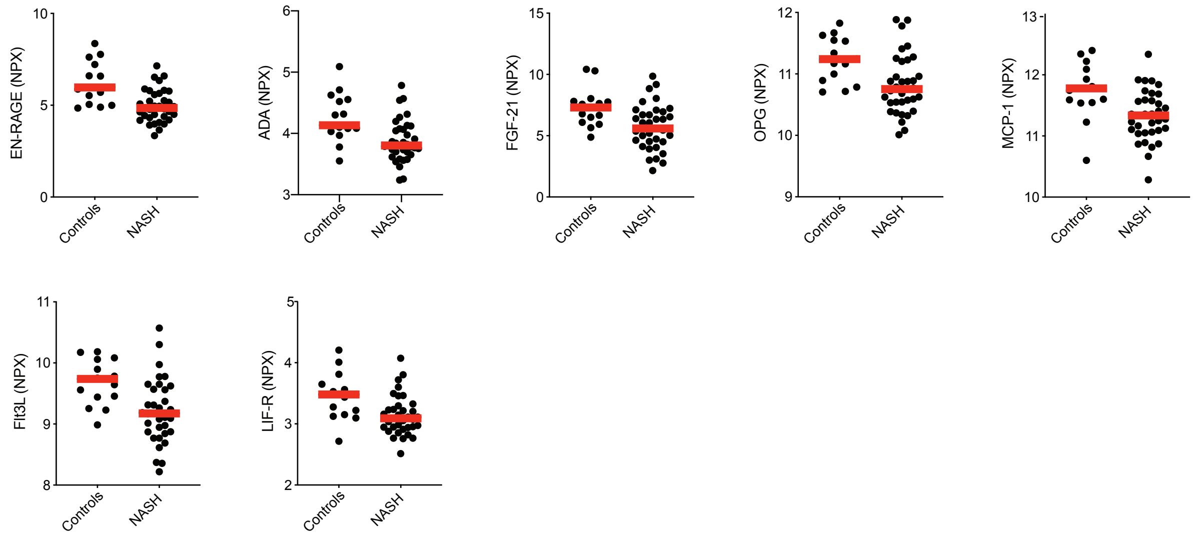

B

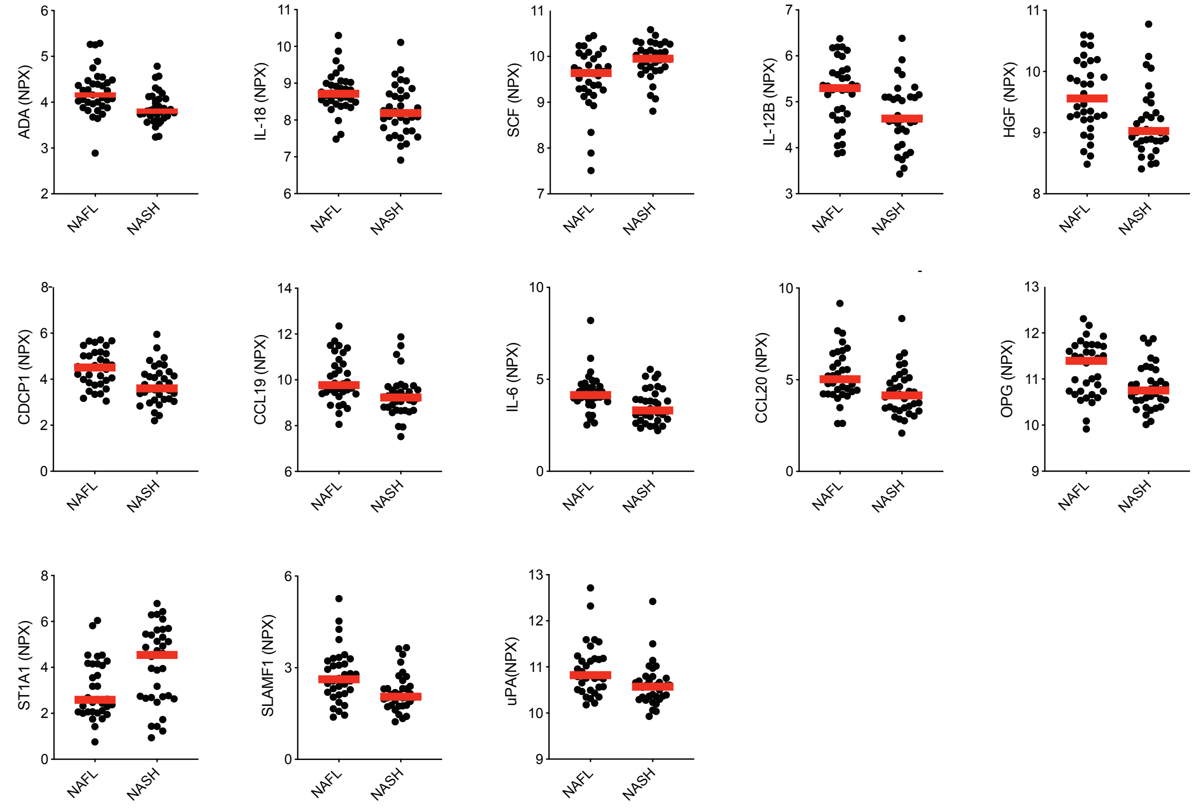

C

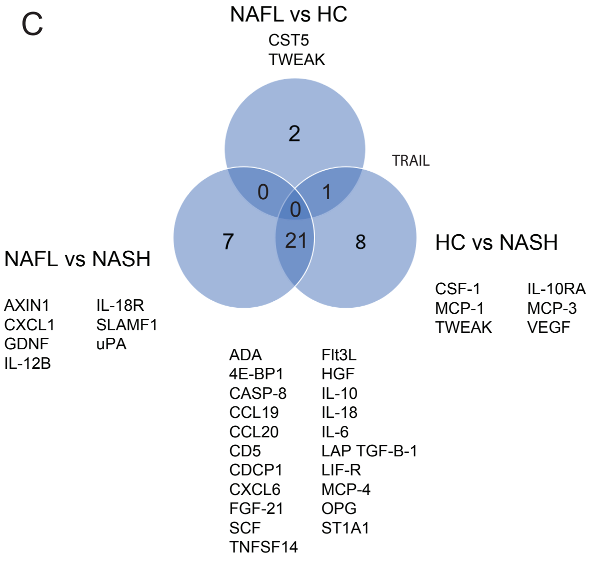

Supplementary figure 4

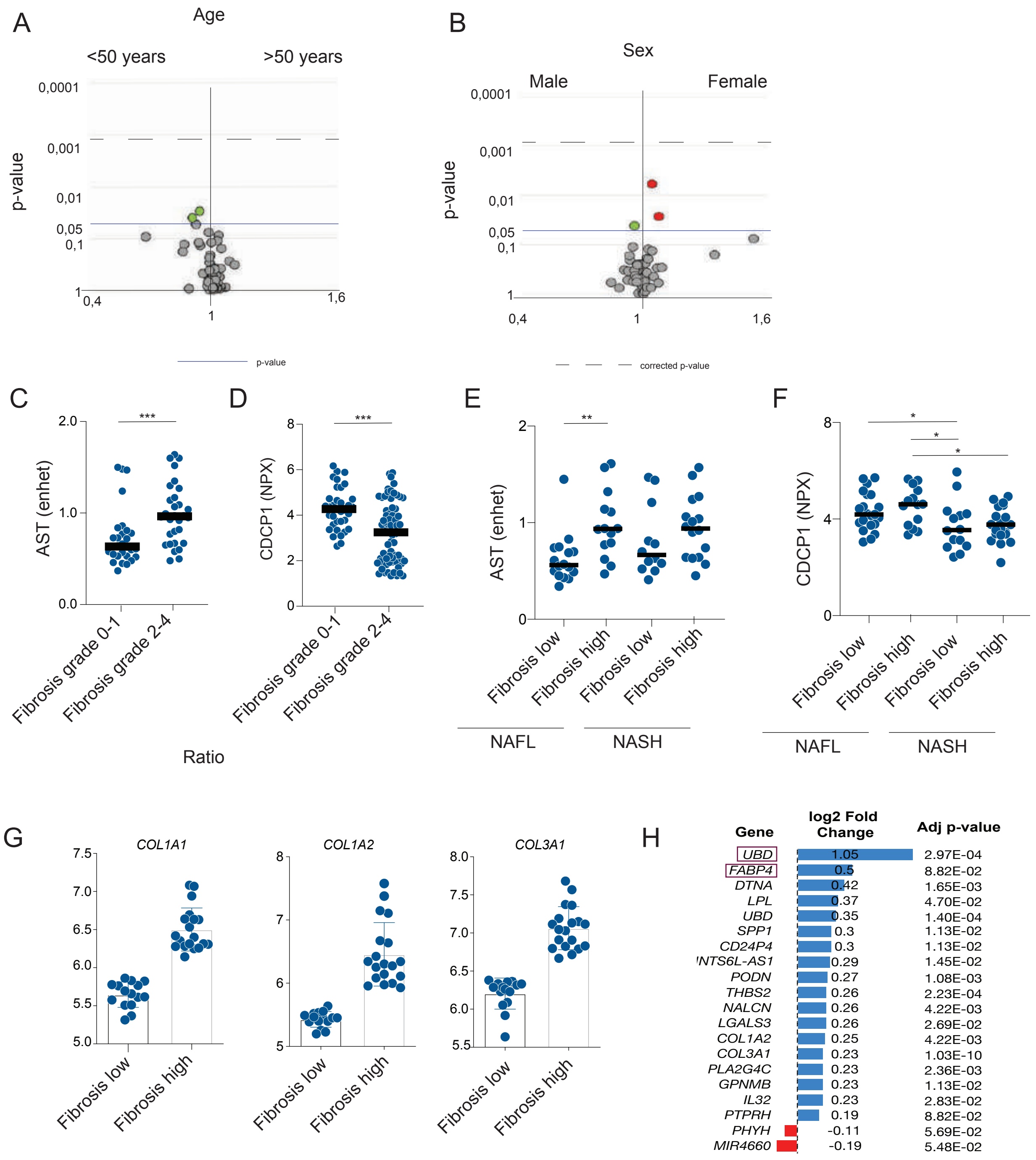

Supplementary figure 5

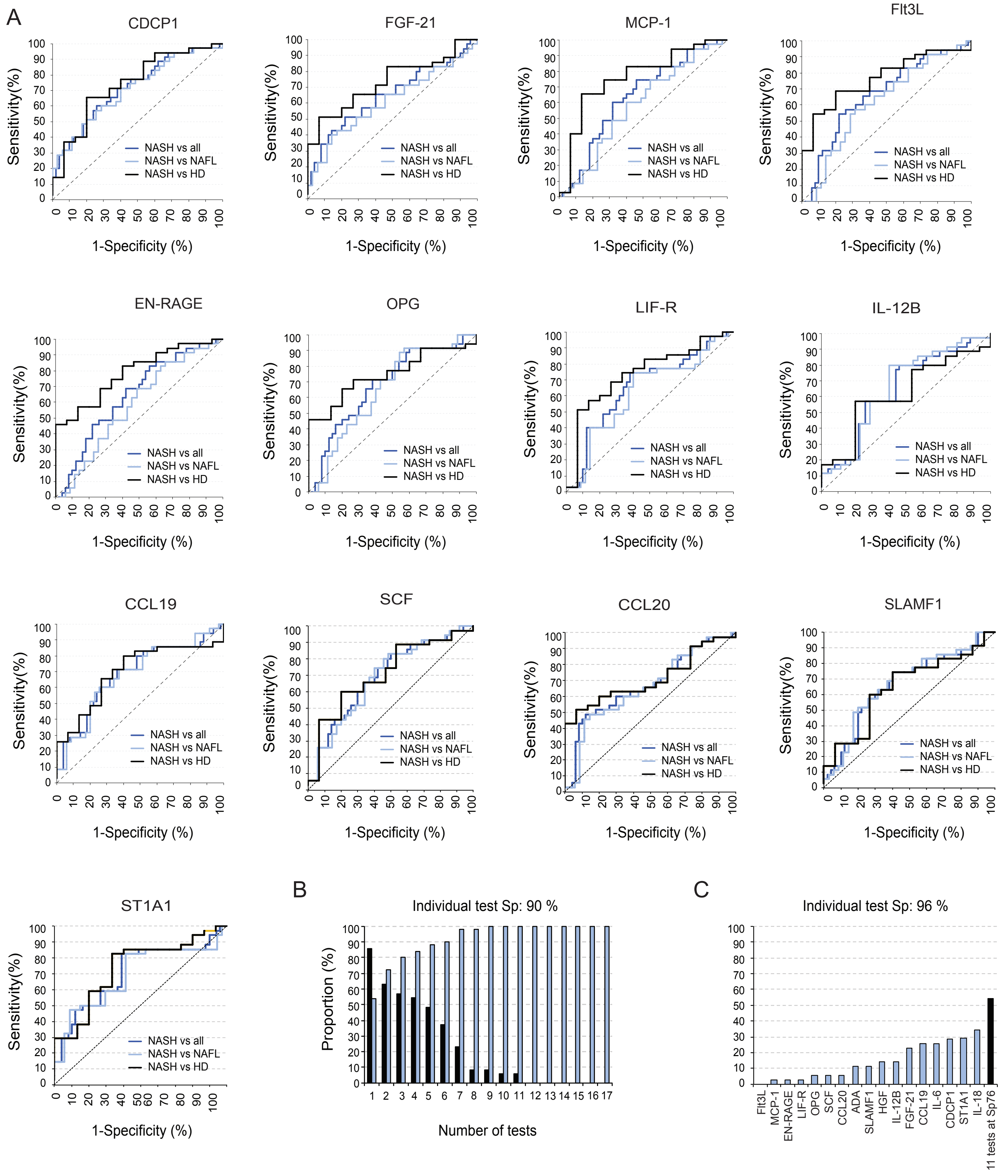

Supplementary figure 6

A

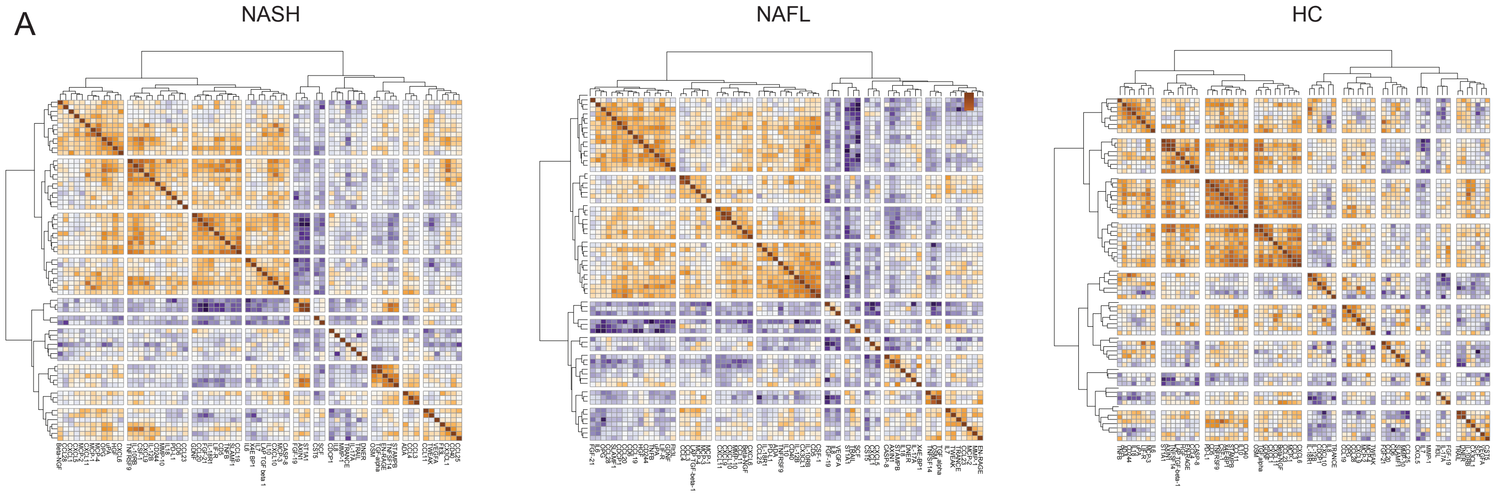

B

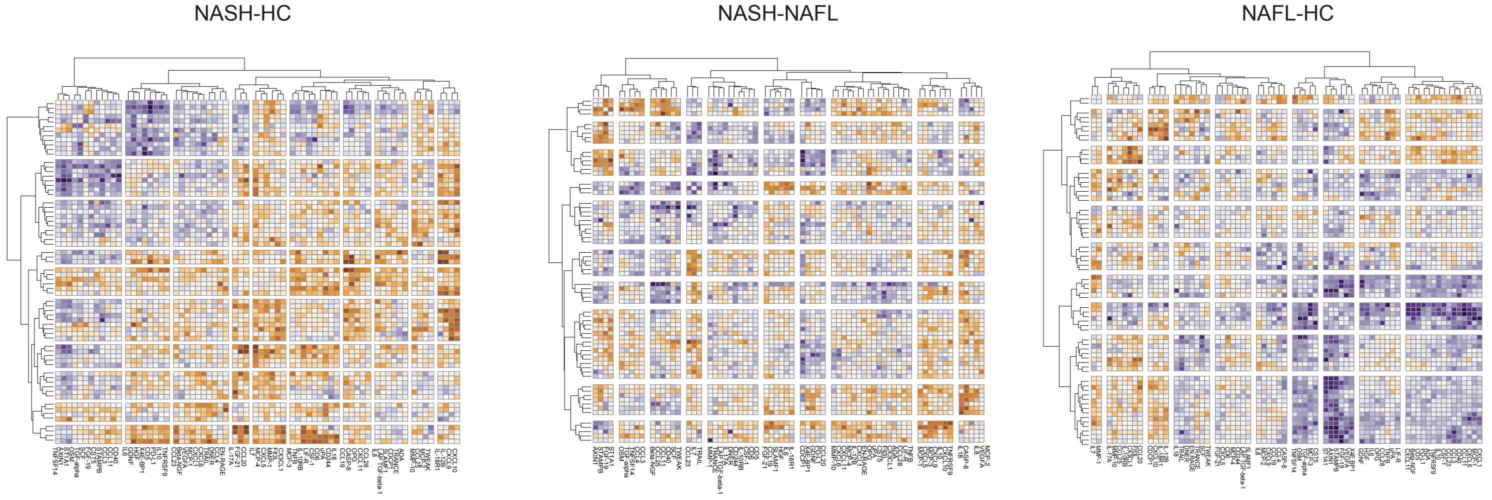

C

NASH-HC

		R difference	p-values	R HD	R NASH	p-values
CASP-8	FGF-21	0,89	0,153	-0,389	0,5039	0,002
CCL19	IL-7	0,88	0,116	-0,425	0,4585	0,0056
CCL20	IL-18R1	0,88	0,576	-0,157	0,7193	<0,0001
CCL20	IL-12B	0,85	0,086	-0,4607	0,384	0,0228
TRANCE	IL-17A	0,83	0,042	-0,536	0,2892	0,2892
CCL20	CCL19	0,83	0,442	-0,21	0,6171	<0,0001
IL-18R1	IL-17A	0,82	0,017	-0,614	0,209	0,234
CCL20	CASP-8	0,82	0,196	-0,354	0,4661	0,0048
CCL20	CD5	0,78	0,458	-0,207	0,5754	0,0003
CASP-8	IL-7	0,77	0,086	-0,461	0,3123	0,0678

NASH-NAFL

		R diffence	p-values	R NAFL	R NASH	p-values
ST1A1	CCL11	0,71	0,0936	-0,2922	0,4173	0,0141
CCL23	OPG	0,67	0,1919	-0,2259	0,444	0,0075
FGF-19	OSM	0,64	0,009	-0,4349	0,2076	0,2315
TNFRSF9	IL-18	0,63	0,5835	0,09595	0,7246	<0,0001
CASP-8	CXCL10	0,60	0,8586	-0,03124	0,5717	0,0003
CASP-8	MCP-1	0,54	0,5835	-0,09595	0,4406	0,0081
FGF-19	TNFSF14	0,54	0,3808	-0,1528	0,3826	0,0233
CASP-8	CXCL11	0,53	0,4911	-0,1203	0,405	0,0158
CASP-8	FGF-21	0,52	0,9103	-0,01975	0,5039	0,002
OPG	IL-7	0,52	0,3834	-0,152	0,3709	0,0283

NAFL-HC

		R difference	p-values	R HD	R NAFL	p-values
CCL20	IL-12B	1,10	0,0861	-0,4607	0,6378	<0,0001
CX3CL1	CXCL10	0,98	0,1607	-0,3821	0,5983	0,0001
TRANCE	IL-17A	0,96	0,0422	-0,5357	0,4258	0,0151
CX3CL1	CDCP1	0,94	0,1262	-0,4143	0,5288	0,0013
IL-17A	CD244	0,91	0,1127	-0,4286	0,4761	0,0059
CCL20	CD5	0,89	0,4578	-0,2071	0684	<0,0001
TNFSF14	IL-7	0,87	0,0562	-0,5071	0,3649	0,0311
CX3CL1	IL-12B	0,87	0,763	-0,08571	0,784	<0,0001
CCL20	CX3CL1	0,86	0,3402	-0,2643	0,5949	0,0002
TNFB	FIt3L	0,86	0,3607	-0,2536	0,605	0,0001

Supplementary figure 7

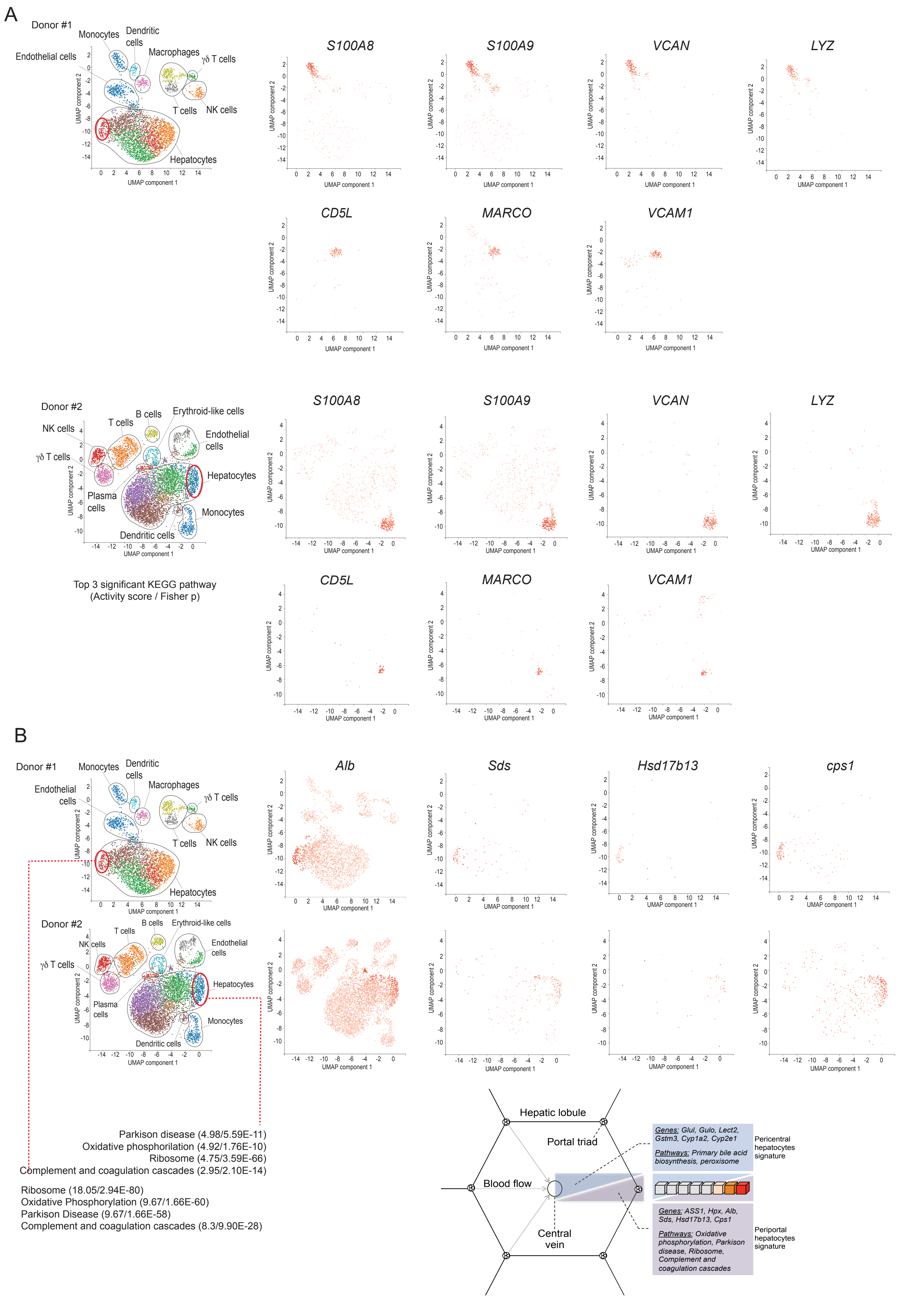

Supplementary figure 8

A

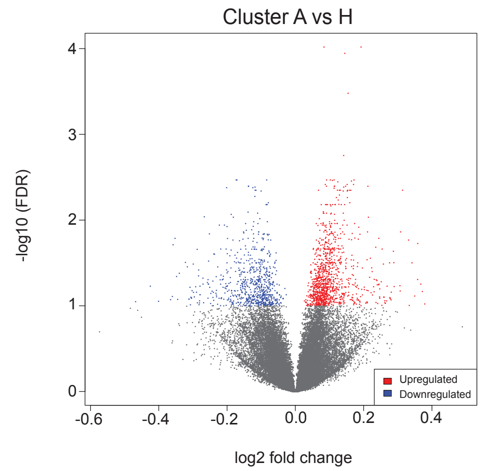

B

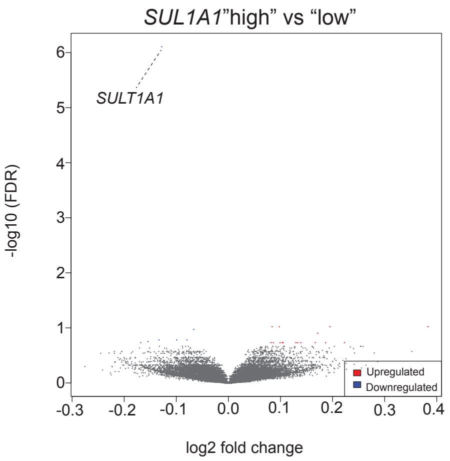

C

Comparison cluster A - F

Adj. Pval	Fold Change	Symbol	Drug
2E-04	0.38	*S100A12*	Amlexanox, Methotrexate
2E-04	0.32	*FPR3*	Pyrazinamide
2E-04	0.30	*S100A8*	Methotrexate
5E-04	0.25	*IL18*	Tracolimus, MYCOPHENOLIC ACID, COLCHICINE,
6E-04	0.24	*ANGPT2*	BEVACIZUMAB, RIBAVIRIN
8E-04	0.22	*DDIT3*	NICOTINE,
6E-04	0.22	*VCAM1*	CARVEDILOL, MERCAPTOPURINE, DEXAMETHAS
5E-04	-0.16	*TNFRSF1A*	CYCLOPHOSPHAMIDE
8E-04	-0.16	*EPHB6*	VANDETANIB, RETINOL, FLUTICASONE PROPION
8E-04	-0.21	*COL11A1*	OCRIPLASMIN
3E-04	-0.24	*TNFRSF8*	BRENTUXIMAB VEDOTIN,

Enriched TF binding motifs in promoters of DEGs, Upstream 300 bp as promotor

List	Motif	TF	TF family	FDR	Drug
Up regulated	TTGGCGCGAAAATTG	E2F1	E2F	4E-14	ASCORBATE, ETOPOSIDE, PACLITAXEL, METHOTREXATE
	GTGACGTAAC	ATF2	bZIP	1E-10	STREPTOZOTOCIN
Down regulated	CCCCGGGC	TFAP2A	AP-2	6E-09	INDINAVIR, AMPRENAVIR, NELFINAVIR,
	GGGAGT	EP300	Unknown	2E-08	METHYLPHENIDATE

Comparison cluster A - B

Adj. Pval	log2 Fold Change	Symbol	Drug
9E-03	0.13	*FECH*	CHOLIC ACID, METHYL AMINOLEVULINATE

List	Motif	TF	TF family	FDR	Drug
Up regulated	CCGGAAG	ELK1	Ets	7E-10	GANCICLOVIR
	CG	MLL	CxxC	2E-07	BELINOSTAT, DOXORUBICIN, CYTARABINE, ROMIDEPSIN,
	TCGCGCCAAA	E2F1	E2F	2E-06	ESTRADIOL, VASOPRESSIN, ASCORBATE, ETOPOSIDE, B
	TTACGTAA	ATF2	bZIP	5E-05	STREPTOZOTOCIN
	CCG	DNMT1	CxxC	7E-05	AZACITIDINE, DECITABINE, FLUCYTOSINE, FLOXURIDINE

Comparison cluster G - F

Adj.Pval	log2 Fold Change	Symbol	Drug
3E-03	0.24	LIPF	ORLISTAT, PALIFERMIN
9E-03	0.24	TNFRSF8	BRENTUXIMAB VEDOTIN, FLUTICASONE PROPIC
7E-03	.07	RALBP1	CARBAMAZEPINE
8E-03	-0.11	UMPS	TEGAFUR
9E-03	-0.13	SLK	DASATINIB
7E-03	-0.17	PSMD1	BORTEZOMIB, CARFILZOMIB, IXAZOMIB CITRATE
5E-03	-0.18	PSMD2	BORTEZOMIB, CARFILZOMIB, IXAZOMIB CITRATE
7E-03	-0.20	ATRX	DOXORUBICIN, VINCRISTINE, TOPOTECAN, TEM

List	Motif	TF	TF family	FDR	Drug
Down regulated	CCGGAAGT	ELK1	Ets	5E-07	GANCICLOVIR
	TTGGCGCGAAAATTG	E2F1	E2F	5E-07	
	TTACGTAA	ATF2	bZIP	1E-05	STREPTOZOTOCIN
Up regulated	GGGGATTCCC	NFKB1	Rel	6E-03	THALIDOMIDE, BORTEZOMIB
	CACCACCTGT	MYOD1	bHLH	7E-03	TAMOXIFEN, TRETINOIN, BUPIVACAINE, LIOTHYRONINE S

D

Comparisons	DEGs	random 1	random 2	random 3	random 4	random 5	random 6	random 7	random 8	random 9	random 10	Median DEGs of 10 random
ClusterA-ClusterF	9087	0	0	0	0	8	1	1	0	0	1	0
ClusterE-ClusterF	4181	0	0	0	0	6	1	1	0	0	0	0
ClusterC-ClusterF	4025	1	1	0	0	1	2	0	0	0	2	0,5
ClusterF-ClusterG	2800	1	5	0	55	10	2	1	5	3	0	2,5
ClusterA-ClusterB	2632	0	0	2	0	0	0	0	0	0	0	0
ClusterA-ClusterH	1555	1	22	4	0	0	2	0	1	12	0	1
ClusterD-ClusterF	409	0	0	2	0	3	0	0	0	2	0	0
ClusterB-ClusterF	400	0	0	0	3	16	2	1	0	0	5	0,5
ClusterF-ClusterH	228	1	2	1	0	0	2	0	1	4	3	1
ClusterE-ClusterH	217	3	17	0	0	0	1	0	0	5	0	0
ClusterB-ClusterG	148	16	50	5	459	21	13	9	9	15	8	14
ClusterB-ClusterH	124	8	31	2	7	2	8	1	1	33	9	7,5
ClusterC-ClusterH	36	2	0	0	0	2	2	0	0	1	0	0
ClusterA-ClusterG	31	0	30	2	207	6	3	1	19	1	2	2,5
ClusterB-ClusterD	28	0	10	23	44	165	0	12	5	17	8	11
ClusterE-ClusterG	24	1	16	2	228	6	1	1	13	3	9	4,5
ClusterA-ClusterD	21	0	14	16	26	1	0	0	1	4	1	1
ClusterG-ClusterH	11	1	1	1	13	2	3	0	2	4	0	1,5
ClusterD-ClusterG	8	0	3	1	13	3	0	0	2	6	0	1,5
ClusterB-ClusterE	8	0	0	0	0	0	2	0	9	1	0	0
ClusterD-ClusterE	7	0	4	2	25	1	3	1	1	24	0	1,5
ClusterC-ClusterG	7	1	0	0	11	7	0	1	4	2	3	1,5
ClusterA-ClusterE	7	0	0	0	0	0	0	0	1	0	0	0
ClusterD-ClusterH	3	0	0	1	0	0	2	0	0	2	0	0
ClusterB-ClusterC	1	13	10	3	60	3	0	0	0	0	0	1,5
ClusterC-ClusterE	0	1	1	0	0	0	0	0	0	0	0	0
ClusterC-ClusterD	0	1	2	2	0	2	0	0	0	3	0	0,5
ClusterA-ClusterC	0	9	1	2	12	3	0	0	0	0	0	0,5
